# An examination of the association between lifetime history of prostate and pancreatic cancer diagnosis and occupation in a population sample of Canadians

**DOI:** 10.1371/journal.pone.0227622

**Published:** 2020-02-05

**Authors:** Smriti Singh, James Ted McDonald, Gabriela Ilie, Anil Adisesh

**Affiliations:** 1 Faculty of Medicine, Dalhousie University, Halifax, Nova Scotia, Canada; 2 University of New Brunswick, Fredericton, New Brunswick, Canada; 3 Department of Community Health and Epidemiology, Urology, and Radiation Oncology, Faculty of Medicine, Dalhousie University, Halifax, Nova Scotia, Canada; 4 Department of Medicine, University of Toronto, Toronto, Ontario, Canada; University of Montana, UNITED STATES

## Abstract

**Background:**

Occupation was assessed as possible risk factors for prostate (PCa) and pancreatic cancer in a large Canadian worker cohort.

**Methods:**

The Canadian Census Health and Environment Cohort (CanCHEC) was derived from linking the 1991 Canadian Census Cohort to the Canadian Cancer Database (1969–2010), Canadian Mortality Database (1991–2011), and Tax Summary Files (1981–2011). From the total sample of 1,931,110 persons, we identified and derived two samples of 28,610 men and 3,220 men and women with a past history of PCa and pancreatic cancer diagnoses, respectively. Cox proportional hazards models were used to estimate hazards ratios and 95% confidence intervals for occupation.

**Results:**

In Canadian men aged 24–64 years, the highest elevated risks of PCa were observed for library clerks (HR = 2.36, 95% CI:1.12–4.97), medical radiation technologists (HR = 1.66, 95% CI:1.04–2.65), telecommunications and line cable workers (HR = 1.62, 95% CI: 1.22–3.16) and commissioned police officers (HR = 1.54, 95% CI: 1.10–2.16. The highest elevated risk for pancreatic cancer were observed for commissioned police officers (HR = 4.34, 95% CI: 1.85–10.21), photographic and film processors (HR = 3.97, 95% CI:1.69–9.34), railway and motor transport labourers (HR = 3.94, 95% CI: 1.67–9.29), and computer engineers (HR = 3.82, 95%CI: 1.52–9.61).

**Conclusion:**

These findings emphasize the need for further study of job-related exposures and the potential influence of non-occupational factors such as screening practices.

## Introduction

Cancer is the leading cause of death in Canada accounting for about 30% of all deaths [1]. Currently, about 1 in 2 Canadians develop cancer in their lifetime, and about 1 in 4 Canadians are estimated to die from it [2]. The number of new cancer cases in Canada is higher among males than females and will as much as almost double among Canadians in the years to come increasing from 80,800 cases in 2003–2007 to 148,400 in 2028–2032 in males and from 74,200 to 128,800 in females [2–3]. These increases are largely due to our aging population and to a lesser extent an increase in population size [3–4]. The current lifetime risk of prostate cancer for men living in Canada is estimated to be approximately one in eight, but incidence is highly dependent on the number of prostate-specific antigen (PSA)-driven biopsies [1,5–7].

Localized prostate cancer (PCa) is the most diagnosed form of cancer among men, with the highest 10 year survival rate (98%) far exceeding the average for other, more aggressive forms of cancer [5–6]. Metastasized PCa has a 5 years survival rate of 30% [3,5]. The estimated incidence of prostate cancer in Canada is 21,300 new cases per year [1]. The highest mortality rates for PCa in 2017 were observed among Prince Edward Island residents at 31.2 deaths per 100,000 varying significantly from British Columbia (BC) which has the lowest mortality rate at 22.2 per 100,000 [1]. Ten year mortality rates for PCa are lowest (about 2%) among all forms of cancer and active forms of treatment (radical prostatectomy, radiation therapy, and hormonal therapy) for both early and late stage disease have seen significant improvements over the years (e.g., robotic-assisted radical prostatectomy, brachytherapy, IMRT) [7–9]. In addition to improvements in active treatments for PCa, low mortality rates have also been attributed to early detection through PSA screening which are easy to administer (blood test screening), and together with physical examinations, highly reliable [2]. PSA testing however, has been criticized because it is often performed in asymptomatic men, where it is believed that many of these subsequently recognised PCa, if left undetected, would never have become clinically meaningful during a man’s lifetime [10]. While active treatments have become very effective at prolonging life, they often lead to urinary (and sometimes bowel) and sexual dysfunction challenging a man’s identity, further leading to emotional and psychological side effects that severely impact the survivor’s quality of life short and long term [11–14].

In contrast, pancreatic cancer is the most aggressive, and 4^th^ leading cause of cancer-related death in Canada and unlike PCa which typically gets diagnosed earlier on, pancreatic cancer is usually diagnosed at a late stage of disease when curative surgical treatments are no longer an option [15]. The estimated incidence of pancreatic cancer in Canada for 2017 is 2,800 for males and 2,700 for females while the five-year survival rate is the lowest of all cancers (7% for males and 8% for females) [15,16]. In 2017, pancreatic cancer accounted for 6% of all cancer related deaths [1,15,16]. Unlike prostate and other forms of cancers, however, pancreatic cancer is relatively unresponsive to chemotherapy and radiation [16,17]. Thus, identifying modifiable risk factors for both these forms of cancer is very important to help implement preventative measures at a population level, diminish their onset, subsequent long term QoL issues (PCa) and high mortality rates (pancreatic cancer).

The development of PCa has several known factors including age, ethnicity, family history, and a diet high in red meat [5,17–19]. Study of age-specific incidence curves reveals that prostate cancer risk begins to rise sharply after 55 years of age, peaks between 70–74 years of age and declines slightly thereafter [18]. African Americans have the highest rates of prostate cancer worldwide with an incidence of 223 per 100,000 [19]. There is also a two to three-fold increased risk of prostate cancer among individuals who have a first degree relative who has been diagnosed with prostate cancer [18–19]. Increased consumption of red meats, fat and dairy products have been associated with an increased risk of prostate cancer in some studies [1,18] while others have shown mixed results [20]. Dietary factors may be implicated in the development of the disease, as obesity has been associated with an almost two times higher risk of prostate cancer in some studies [21] while others have shown that obesity in itself is not a risk factor, but is associated with a poorer prognosis [22,23]. Sexually transmitted infections and sexual frequency have been implicated as an increased risk for prostate cancer with syphilis posing the greatest relative risk at 2.3 (95% CI:1.3–3.9) and sexual frequency of greater than two times per week being associated with a relative risk of 1.2 (95% CI: 1.1–1.3) [24].

Investigating occupation as a risk factor for prostate cancer in a Montreal study, Sauvé, Lavoué and Parent (2016) reported excess prostate cancer risk among forestry workers, policemen, some predominantly white collar occupations, and industries such as public service, administrative and clerical work [25]. Earlier, Parent and Siemiatycki concluded that it is plausible that certain pesticides or herbicides, act as hormone modifiers to influence prostate cancer risk. They also considered that experimental evidence of cadmium carcinogenicity meant further attention to possible human carcinogenic effects was warranted [26]. Certain metal exposures, e.g. lead, cadmium, hexavalent chromium compounds and arsenic, are considered relevant prostate carcinogens by Doolan, Benke and Giles with their 2014 review stating, “there is sufficient evidence to implicate toxic metals, polychlorinated biphenyls and polycyclic aromatic hydrocarbons” [27] as well as other authors [21,25,28]. Several studies have also found associations with industries such as forestry, firefighting, police and rubber production, particularly with the last two occupations showing an increased risk of prostate cancer [25,28,29].

There have been some modest, but statistically significant decreases in mortality rates for pancreatic cancer in Canada, a decrease of approximately 0.6% per year for men and 0.4% for women between 1992 and 2012 [15, 16]; however, it is not known what has led to these decreases, as results vary across other countries such as the United Kingdom and the United States [30]. Our poor ability to detect and treat pancreatic cancers indicate that preventative measures may be the best method to curb the high mortality rate of this disease.

The development of pancreatic cancer has several known risk factors including family history and genetic disorders, hormonal factors, comorbidities, cigarette smoking, excessive alcohol consumption, obesity, diet, and occupational exposures [30–36]. Family history of pancreatic cancer in a first degree relative is associated with an increased risk of pancreatic cancer of 2.5 to 5.3 times [30]. The risk increases with more relatives affected and it persists even when adjusting for smoking within the household [30]. There are also several genetic disorders associated with an increased risk of pancreatic cancer including pancreatitis [30,32] familial atypical multiple mole melanoma [33], Peutz-Jeghers syndrome [37], and cystic fibrosis [34]. Patients with hereditary pancreatitis have a 53-times (95% CI: 23–105) greater risk of disease with a cumulative lifetime risk of 40% of developing pancreatic cancer [30,31].

There are several studies in females which have shown a protective association with parity in the development of pancreatic cancer [30,38]. A potential mechanism may be from the decreased insulin-like growth factor and decreased iron stores during the time of pregnancy. It is believed that insulin-like growth factor promotes cell proliferation and inhibits apoptosis, while increased iron stores can induce carcinogenesis by promoting DNA damage through oxidative stress [30].

Diabetes mellitus [35], acute pancreatitis [30] and chronic pancreatitis [32] have been shown to increase the risk for pancreatic cancer. Diabetes mellitus is associated with an odds ratio of 1.82 (95% CI: 1.66–1.89), while several studies have found a relative risk of 2.2 (95% CI: 1.6–2.9) and 5 (95% CI: 4.1–6.1) for any form of pancreatitis [30,31]. Currently, cigarette smoking (increased usage for longer periods of time showing greater risk), alcohol and obesity are the only identified modifiable risk factor for pancreatic cancer [30]. Some studies have estimated that the risk for developing pancreatic cancer is almost double (OR = 1.74; 95% CI: 1.61,1.87) among smokers compared to non-smokers [24]. Other lifestyle factors which have been found to have mixed associations with pancreatic cancer include alcohol use, BMI and diet [30,31]. One study, completed in 2013 observed an increased risk of pancreatic cancer in those who consume greater than 6 alcoholic beverages per day (OR = 1.46, 95% CI: 1.16–1.83) and for those with BMI greater than 35 (OR = 1.55, 95% CI: 1.16–2.07) [16].

Occupational exposures have been examined for over 20 different agents and found to be contributing. Among the agents examined, chlorinated hydrocarbon solvents have shown an increased meta-risk of 1.4 (95% CI: 1.0–1.8), whereas nickel and nickel compounds have shown a meta-risk of 1.9 (95% CI: 1.2–3.2) [37, 39]. Other compounds such as cadmium, chromium compounds, polycyclic aromatic hydrocarbons, organochlorine insecticides, pesticides, silica dust, asbestos and metalworking fluids have produced mixed results [30,39,40,41].

An estimated 50–60% of PCa have an inherited component, with the most common inherited genetic alteration being the BRCA2 gene, which is also linked with increased risk for pancreatic cancer [42]. It is believed that fewer than 10% of cancers arise from germline mutations, meaning that the majority of cancers are acquired over the course of an individual’s lifetime [43]. This is evidenced by studies conducted on adopted children and their tendency to acquire the cancer risk of their adoptive rather than biological parents’ risks; as well as studies of migrant populations adopting cancer risks similar to their place of residence rather than their birth country [44]. Such evidence points to the importance environmental factors play in the development of cancers and how these influences cannot be ignored. In order to better understand modifiable factors in the development of cancer, the International Agency for Research on Cancer (IARC) has identified more than 900 agents and classified more than 400 known or suspected carcinogens [45,46]. Of these, 168 individual agents and 18 exposure situations are found in occupational environments [47]. Since occupation plays a large role in an individual’s lifetime, both in terms of hours spent at the workplace and exposure during work hours to certain materials and compounds that may predispose to cancer onset, occupation is a significant environmental factor in the development of certain cancers. As progress continues to be made in both survival and mortality rates for prostate and pancreatic cancer, identifying risk factors for each could help lead to better methods of primary prevention, alleviating burden on tertiary care centers.

Early research on occupationally related cancer burden estimated as much as 20% of total cancer mortality [47]. A more recent study completed in Great Britain in 2010, examined relevant IARC agents which were deemed “established” or “probable” carcinogens and found occupationally related cancers responsible for 5.3% of total cancer burden [46,47]. A recently released Canadian report on the Occupational Cancer Burden estimates that in 2011 there were 9,700 to 10,400 cases of cancer attributable to past occupational exposure representing 3.9 to 4.2% of the total cases [48]. Whilst this report identifies important occupational carcinogens it does not address individual cancer or occupational estimates. However, smaller, exposure specific studies have implicated a link between certain cancers or occupations [21,25,28,30,39–41]. A recent study by Sritharan et al. (2018) found elevated risks of PCa incidence in occupations related to administration and management, agriculture, and protective services and decreased risks in construction and transportation in a population-based sample of Canadian men aged 25–74 years. They also observed an elevated risk of PCa mortality among agriculture management workers. The current study expands Sritharan et al. (2018) study by examining the hazard risk of prostate, but also pancreatic cancer within occupation industries and occupation titles in a population based sample of Canadians ages 25 to 64, while controlling for age, sex, household income, education level, immigration status, province of residence to determine whether certain occupations have a statistically significant increased risk of either form of cancer. Examining prostate and pancreatic cancers risk across occupational categories can also serve as a substitute for exposure assessment, given some evidence indicating a common inherited genetic alteration for these two types of cancer [42], and help determine areas where prevention research should focus.

## Methodology

### Study cohort

Our analytic sample was based on a subsample of 2,644,370 Canadian adult men and women surveyed in 1991 as part of the Canadian Census Health and Environment Cohort (CanCHEC) 1991. Data was accessed through the New Brunswick Statistics Canada Research Data Centre in Fredericton. The CanCHEC contains several databases including the 1991 Canadian Long-form Census, 1984–2011 Historical Tax Summary Files, 1981–1983 T1 Personal Master File, 1969–1991 Canadian Cancer Database, 1992–2010 Canadian Cancer Registry and 1991–2011 Canadian Mortality Data Base [49].

The Canadian long form census is a mandatory survey conducted every 5 years in Canada and is administered to 20% of the Canadian population. The 1991 long form census was administered June 4, 1991. The study population included Canadian individuals between 25–64 years of age on census day, which resulted in a sample size of 2,644,370. This age range was chosen on the basis that the average age of retirement for Canadians has consistently been around 62 years of age [50] and that cancers diagnosed in this period would be more representative of occupational exposures than other lifestyle or age-related factors. One additional reason for the age range is that occupation is likely to be measured with significant error or be missing for those currently retired. Income and education level were chosen as control variables because they were found previously to correlate with smoking and other lifestyle factors. They also can exert an independent effect on cancer since they proxy for a whole range of other unobserved factors such as social status, employment stability, better general health and others. We also note that socioeconomic status in investigations of the type we present here is typically measured by area level average income, whereas we include individual measures of socioeconomic status.

One Canadian study noted that compared to university graduates, those with a secondary school education or less had three times the odds of being a current smoker and those with secondary or college degrees only, had a double or more odds of being a current smoker [51]. Similarly, household income has been found to be correlated to obesity with one Canadian report citing a 0.76% and 0.27% decrease in obesity for men and women, respectively for every 1% increase in household income [52]. By adjusting for these factors, socioeconomic differences can be accounted for within the models and the role socioeconomic status plays in the development of cancer.

**Fig 1:**
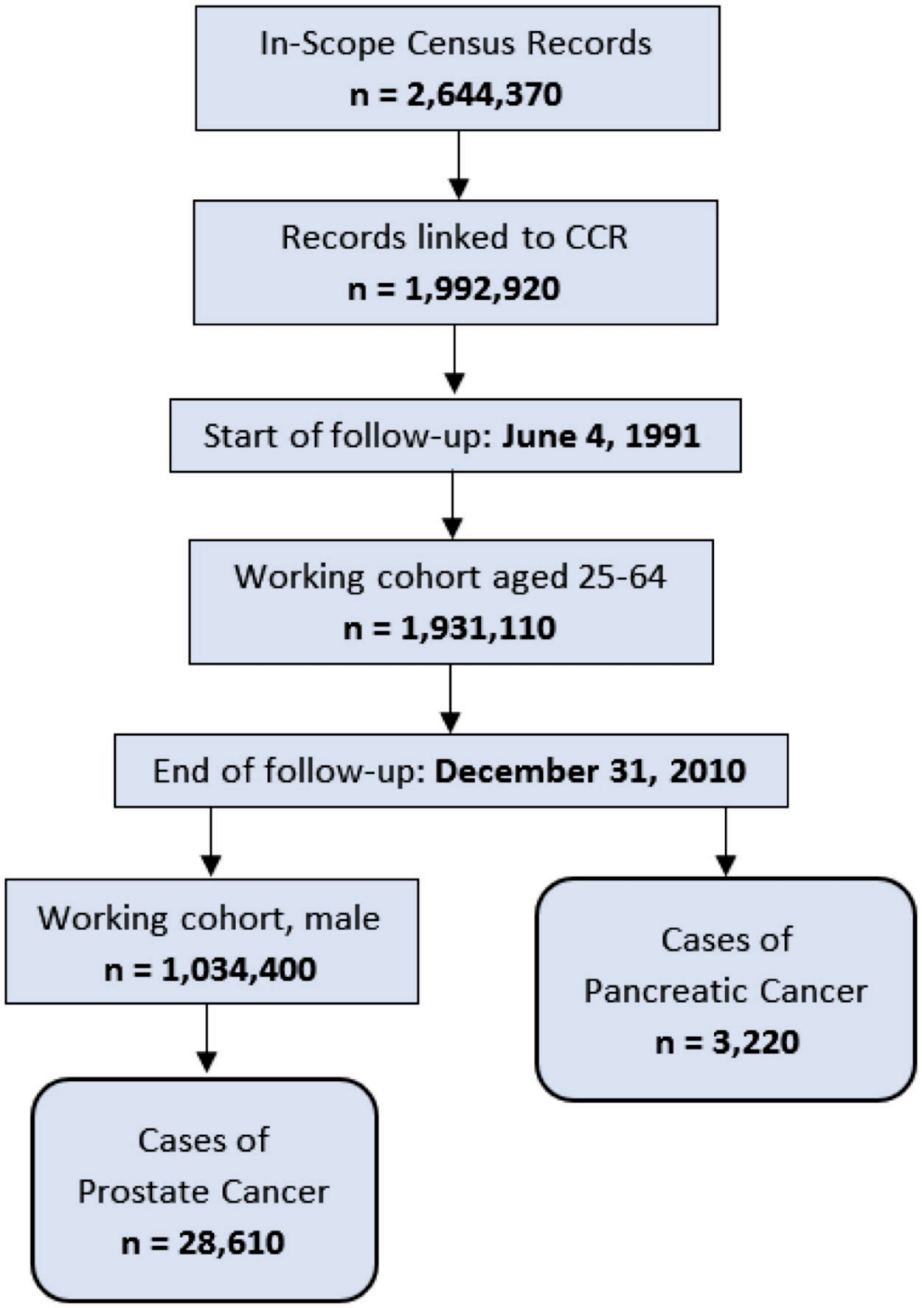
Derivation and linkage of prostate and pancreatic cancer study cohort using the 1991 CanCHEC dataset.

The study end date was December 31^st^, 2010, which corresponded to the end records of the Canadian Cancer Registry at the time this study was conducted. We censored observations if the individual had multiple primary cancers, or primary cancer was not prostate or pancreatic cancer, if the individual stopped filing taxes within the study period or if the individual died of another cause during the study period. After applying the exclusion criteria and linking the databases the in-scope sample size of the working cohort was 1,931,110 including 3,220 cases of pancreatic cancer. The working male cohort included 1,034,400 males with 28,610 cases of prostate cancer. Fig 1 displays how the data was linked and how the study cohort was obtained. This study complied with the University of New Brunswick Institutional Review Board ethics requirements, which do not require an additional review for research projects using Statistics Canada data stored in the NB-RDC.

Occupational history was determined by 1990 National Occupational Classification (NOC) coding. Occupation is classified using this method in the long form census by the kind of work an individual was completing during the reference week, as determined by the description of their most important job duties. Data is available for persons 15 years of age and over, excluding institutional residents. If a person did not have a job during the week prior, then the data related to the job of longest duration since January 1, 1990 is recorded. For persons with two or more jobs, the job with the most hours is recorded. Within this classification, the coding structure has a 4-digit coding, revisions to the NOC have taken place periodically with relatively minor changes but with an explicit four-tiered hierarchical structure based on the earlier 4-digit codes (S1 Table). We aggregated the 4-digit 1990 NOC codes to the 2-digit coding used by the 2011 NOC allowing the representation of broad occupational groupings e.g. “Senior management occupations” is coded as NOC 2011 2-digit (“00”coding and within this the NOC 1990 4-digit coding “0012” represents a specific job title “senior government managers and officials”.

### Statistical analysis

Statistical analysis was completed using Stata v14. Hazard ratios and corresponding 95% confidence intervals were calculated using Cox Proportional hazard regression models to estimate prostate or pancreatic cancer risk associated with employment by occupation. A baseline control group was chosen based on a large occupational group which contained a heterogeneous sample of individuals (secondary school teachers, in age category 55–64, n = 26,885). The incidence rate for this group of individuals is very similar to the population incidence which furthers their use as a baseline control group. The baseline control group becomes the reference group for which the odds ratio for other occupations is then estimated. Models also adjusted for age, sex, education level, income, immigration status, and province of residence. The study population was analysed by 4-digit NOC 1990 and 2-digit major group NOC 2011 codes. Individual occupational categories were excluded if 5 or fewer than 5 cases were noted.

## Results

### Prostate cancer

A total of 28,610 cases of history of prostate cancer were identified between 1991 and 2010 in the working cohort of Canadian men aged 24–64 years. As seen in Table 1 an elevated risk of prostate cancer was observed across several broad occupational categories including specialized middle management occupations (HR = 1.13, 95% CI: 1.04–1.24), senior management occupations (HR = 1.12, 95% CI: 1.05–1.19) and business, finance and administration occupations (HR = 1.10–95% CI: 1.01–1.21).

**Table 1:** Hazard ratios and 95% confidence intervals for prostate cancer in CanCHEC (1991–2010) by occupational major group (NOC 2011 2 digit codes) adjusted for age, sex, education level, income, immigration status, and province of residence^1^^,^^2^.

HR	95% CI	Men with history of PCa	Total number of men in the occupational group	Occupational category
**1.13**	1.04–1.24	580	13,460	Specialized middle management occupations
**1.12**	1.05–1.19	1,875	45,960	Senior management occupations
**1.10**	1.01–1.21	615	18,375	Professional occupations in business, finance and administration occupations

^1^ Reference group: Secondary school teachers, HR = 1

^2^ Case counts are rounded to base ten using random rounding and case counts <5 are not shown as per Statistics Canada guidelines

Table 2 displays significant elevated risks observed across several occupation titles including library clerks (HR = 2.36, 95% CI: 1.12–4.97), medical radiation technologists (HR = 1.66, 95% CI: 1.04–2.65), telecommunications and line cable workers (HR = 1.62, 95% CI: 1.22–2.16), commissioned police officers (HR = 1.54–95% CI: 1.10–2.16), and insurance real estate and financial brokerage managers (HR = 1.21, 95% CI: 1.02–1.44).

**Table 2:** Hazard ratios and 95% confidence intervals for prostate cancer in CanCHEC (1991–2010) by occupation (NOC 1990 4 digit codes) adjusted for age, sex, education level, income, immigration status, and province of residence^1^^,^^2^.

HR	95% CI	Men with history of PCa	Total number of men in the occupation	Occupation title
**2.36**	1.12–4.97	5	160	Library clerks
**1.66**	1.04–2.65	20	430	Medical radiation technologists
**1.62**	1.22–2.16	50	1280	Telecommunications and line cable workers
**1.54**	1.10–2.16	35	505	Commissioned police officers
**1.21**	1.02–1.44	160	3120	Insurance, real estate and financial brokerage managers

^1^ Reference group: Secondary school teachers, HR = 1

^2^ Case counts are rounded to base ten using random rounding and case counts <5 are not shown as per Statistics Canada guidelines

Evaluation of province of residence revealed significant elevated risk for prostate cancer individuals residing in New Brunswick (HR = 1.10, 95% CI: 1.03–1.18) and for those within the highest education bracket which included those with a university degree higher than a bachelor’s (HR = 1.10, 95% CI:1.03–1.15).

### Pancreatic cancer

Table 3 displays significant elevated risk for history of pancreatic cancer observed across several broad occupational categories coded at the NOC 2011 2 digit level these include processing, manufacturing and utilities supervisors (HR = 1.65, 95% CI: 1.17–2.32), specialized middle management occupations (HR = 1.63, 95% CI: 1.22–2.18), technical occupations related to natural and applied science (HR = 1.50, 95% CI: 1.16–1.95), technical occupations in art, culture, recreation and sport (HR = 1.49, CI: 1.05–2.13), assisting occupations in support of health services (HR = 1.49, 95% CI: 1.05–2.10), senior management occupations (HR = 1.48, 95% CI: 1.19–1.84), middle management occupations in retail and wholesale trade and customer service (HR = 1.48, 95% CI: 1.20–1.84), and traders, helpers, construction labourers and related occupations (HR = 1.47, 95% CI: 1.09–1.99). Other installers, repairers and servicers and material handlers + transport and heavy equipment operation and related maintenance occupations (HR = 1.47, 95% CI: 1.18–1.83), Professional occupations in natural and applied sciences (HR = 1.45, 95% CI: 1.12–1.88), Workers in natural resources, agriculture and related production (HR = 1.42, 95% CI: 1.04–1.93), Sales support occupations + service support and other service occupations (HR = 1.40, 95% CI: 1.14–1.73), Industrial, electrical and construction trades (HR = 1.39, 95% CI: 1.12–1.72), processing and manufacturing machine operators and related production workers (HR = 1.36, 95% CI: 1.09–1.70), administrative and financial supervisors and administrative occupations + finance, insurance and related business administrative occupations (HR = 1.34, 95% CI: 1.09–1.64), sales representatives and salespersons = wholesale and retail trade + service representatives and other customer and person services occupations (HR = 1.28, 95% CI: 1.04–1.57).

**Table 3:** Hazard ratios and 95% confidence intervals for pancreatic cancer in CanCHEC (1991–2010) by occupational major group (NOC 2011 2 digit codes) adjusted for age, sex, education level, income, immigration status, and province of residence^1^^,^^2^.

HR	95% CI	Individuals with History of Pancreatic Cancer	Total number of individuals in the occupational group	Occupational category
**1.65**	1.17–2.32	40	15,800	Processing, manufacturing and utilities supervisors
**1.63**	1.22–2.18	60	23,030	Specialized middle management occupations
**1.50**	1.16–1.95	80	45,920	Technical occupations related to natural and applied sciences
**1.49**	1.05–2.13	35	21,845	Technical occupations in art, culture, recreation and sport
**1.49**	1.05–2.10	35	22,090	Assisting occupations in support of health services
**1.48**	1.19–1.84	150	64,445	Senior management occupations
**1.48**	1.20–1.84	180	89,440	Middle management occupations in retail and wholesale trade and customer service
**1.47**	1.09–1.99	50	28,975	Traders helpers, construction labourers and related occupations
**1.47**	1.18–1.83	210	95,730	Other installers, repairers and servicers and material handlers + transport and heavy equipment operation and related maintenance occupations
**1.45**	1.12–1.88	80	51,435	Professional occupations in natural and applied sciences
**1.42**	1.04–1.93	50	26,085	Workers in natural resources, agriculture and related production
**1.40**	1.14–1.73	235	126,640	Sales support occupations + service support and other service occupations
**1.39**	1.12–1.72	215	111,870	Industrial, electrical and construction trades
**1.36**	1.09–1.70	185	103,135	Processing and manufacturing machine operators and elated production workers
**1.34**	1.09–1.64	230	152,465	Administrative and financial supervisors and administrative occupations + finance, insurance and related business administrative occupations
**1.28**	1.04–1.57	250	168,525	Sales representatives and salespersons–wholesale and retail trade + service representatives and other customer and personal services occupations

^1^ Reference group: Secondary school teachers, HR = 1

^2^ Case counts are rounded to base ten using random rounding and case counts <5 are not shown as per Statistics Canada guidelines

Elevated risk for pancreatic cancer was also associated with being male (HR = 1.33, 95% CI: 1.23–1.44) and having lower education, such as having no formal education (HR = 1.20, 95% CI: 1.02–1.40) compared with having had a trades certificate or diploma (HR = 1.16, 95% CI: 1.00–1.35).

Table 4 displays specific occupation titles coded at the NOC 1990 4-digit level showing significant elevated risk observed across several jobs with a 2 times or higher risk seen in the following occupations: commissioned police officers (HR = 4.34, 95% CI: 1.85–10.21), photographic and film processors (HR = 3.97, 95% CI: 1.69–9.34), railway and motor transport labourers (HR = 3.94, 95% CI: 1.67–9.29), computer engineers (HR = 3.82, 95% CI: 1.52–9.61), mechanical assemblers and inspectors (HR = 3.14, 95% CI: 1.52–6.47), nursery and greenhouse workers (HR = 2.73, 95% CI: 1.49–5.01), pharmacists (HR = 2.68, 95% CI: 1.36–5.30), mechanical engineering technologists and technicians (HR = 2.64, 95% CI: 1.12–6.22), typesetters and related occupations (HR 2.53, 95% CI: 1.00–6.40), dry cleaning and laundry occupations (HR = 2.52, 95% CI: 1.46–4.35), construction inspectors (HR = 2.28, 95% CI: 1.03–5.09), other elemental service occupations (HR = 2.22, 95% CI: 1.04–4.74), supervisors, mineral and metal processing (HR = 2.13, 95% CI: 1.04–4.38), painters and decorators (HR = 2.03, 95% CI: 1.14–3.63).

**Table 4:** Hazard ratios and 95% confidence intervals for pancreatic cancer in both sexes in CanCHEC (1991–2010) by occupation (NOC 1990 4 digit codes) adjusted for age, sex, education level, income, immigration status, and province of residence^1^^,^^2^.

HR	95% CI	Individuals with History of Pancreatic Cancer	Number of individuals in the Occupation	Occupation title
**4.34**	1.85–10.21	5	515	Commissioned police officers
**3.97**	1.69–9.34	10	1225	Photographic and film processors
**3.94**	1.67–9.29	5	825	Railway and motor transport labourers
**3.82**	1.52–9.61	5	1380	Computer engineers
**3.14**	1.52–6.47	10	1700	Mechanical assemblers and inspectors
**2.73**	1.49–5.01	15	2985	Nursery and greenhouse workers
**2.68**	1.36–5.30	5	2670	Pharmacists
**2.64**	1.12–6.22	20	3730	Mechanical engineering technologists and technicians
**2.53**	1.00–6.40	5	1355	Typesetters and related occupations
**2.52**	1.46–4.35	5	1685	Dry cleaning and laundry occupations
**2.28**	1.03–5.09	10	1810	Construction inspectors
**2.22**	1.04–4.74	15	4020	Other elemental service occupations
**2.13**	1.04–4.38	10	2635	Supervisors, mineral and metal processing
**2.03**	1.14–3.63	15	9340	Painters and decorators

^1^ Reference—group: Secondary school teachers, HR = 1

^2^ Case counts are rounded to base ten using random rounding and case counts <5 are not shown as per Statistics Canada guidelines.

## Discussion

Results we report here for Prostate Cancer closely mirror those of a recent study looking at occupation and title and its links with history of prostate cancer diagnosis by Sritharan et al. [29]. Slight differences in the models we report here and Sritharan et al. [29] include the narrower age range (our selected age range was 25–64 in keeping with the most accurate working aged population, compared to the 25–74 age range used previously), controlling for income level (proxy for social economic status) and therefore isolating factors that are specific to occupation (omitted in the Sritharan et al. investigation), and looking at both 2-digit (broad occupation groups, omitted in the Sritharan et al. investigation) and 4 digit occupation groups (surrogate for more homogeneous/specific exposure when we don’t have an exposure metric). These differences may account for the slight variations in results between the two analyses. The close replication between our studies also instils further confidence in the models we used for assessing hazard ratios for the pancreatic cancer sample we analyzed. Also, looking at both 2 digit and 4-digit occupation groups is important as the latter has more precision in terms of occupation but can also be affected by smaller cell counts and so less robust results.

### Prostate cancer

Sritharan et al. [29] reported an increase risk of history of prostate cancer diagnosis for senior and government management (HR = 1.12, 95% CI: 1.04–1.20), as well as for finance managers and financial services (HR = 1.09, 95% CI: 1.04–1.14) which mirror our results (HRs = 1.12 and 1.10, respectively). Senior and government management jobs are recognized as having few chemical exposures except for printed ink, air and water quality issues from exposure to older building infrastructure. Thus these findings may reflect other environmental factors such as lack of physical activity (e.g., sitting for prolonged periods of time), socioeconomic factors (affording foods that are expensive and are known risks factors for PCa such as red meats; and smoking regularly which have also been linked with increased risk of PCa), and/or diet (e.g., mostly red meats and minimum leafy greens). Future studies should further investigate how these occupations may interact with socioeconomic factors, diet and physical activity to predispose men to PCa.

Sritharan et al. [29] also found that commissioned police officers had an elevated risk of PCa, HR = 1.22, 95% CI: 1.09–1.36) which is slightly lower than the one observed here (HR = 1.54, 95% CI: 1.22–2.65). This finding has also been noted in several other studies, where protective services are observed to have an elevated risk of PCa [25,29], and whilst not a new finding it is intriguing that several different studies are replicating the result. Police officers may be exposed to chemicals e.g. vehicle exhausts, Lead in dust from firing ranges, as well as contagious and infectious diseases. This comes in addition to disruption of circadian rhythms due to irregular shift work, as well as in addition to irregular eating habits, poor diet, and prolonged hours standing, sitting in the car or behind a desk. These results point to important opportunities for PCa prevention and awareness programs in these occupations. These industries could benefit from having PCa awareness sessions where these risk factors could be discussed and solutions to how to minimize their impact could be put forward for those at risk.

Another similarity noted was of significant risk of history of PCa for those in the highest education bracket (Sritharan et al., HR = 1.22, 95% CI:1.19,1.26; current results observed a HR = 1.09, 95% CI:1.03,1.15). Higher education level is a proxy for higher socioeconomic status which could play a role in increased screening leading to earlier diagnosis [29].

New Brunswick residents had also statistically significant increase of risk of history of PCa compared to the other provinces, which may also be a result of differences in screening programs within provinces. Although Canada has universal healthcare other barriers to accessibility may be present, especially for those residing in more rural settings such as New Brunswick. Additionally, the population of individuals aged 65 and older tends to be higher than the national average [50] which may play an important role on education and screening.

Lastly Sritharan et al. found a non-significant decrease risk of prostate cancer for primary metal production. The current manuscript finds the occupational group mineral and metal processing to have an elevated risk (HR = 2.13, 95% CI: 1.04–4.38). This is not surprising as previous literature suggests that cadmium, arsenic and other minerals may increase the risk of prostate cancer whilst there is also exposure to polycyclic aromatic hydrocarbons in these occupations. One possible explanation for the discrepancy between the two studies could be the age group. Perhaps by narrowing our age group the current study was able to identify this association, while with a larger and more heterogeneous age group this association could not have been captured because these individuals may have died, and/or they may not have been representative of the disease or the occupational exposure. Other complexities such as social inequalities in cancer risk that were not accounted for in this study may be at play. Future studies may want to look at populations within populations of socially disadvantaged individuals to see if they could explain this discrepancy.

### Pancreatic cancer

A range of occupational categories and occupation titles showed increased risk for history of pancreatic cancer, including processing, manufacturing and utilities supervisors, specialized middle management occupations, technical occupations related to natural and applied sciences, technical occupations in art, culture, recreation and sport, assisting occupations in support of health services, senior management occupations, middle management occupations in retail and wholesale trade and customer service, traders helpers, construction labourers and related occupations, other installers, repairers and servicers and material handlers and transport and heavy equipment operation and related maintenance occupations, professional occupations in natural and applied sciences, workers in natural resources, agriculture and related production, sales support occupations and service support and other service occupation, industrial, electrical and construction trades, processing and manufacturing machine operators and elated production workers, as well as sales representatives and salespersons–wholesale and retail trade and service representatives and other customer and personal services occupations. To our knowledge this is the first study to profile the risk of pancreatic cancer by occupation to the extent we present here, therefore we don’t have much literature to which to compare the current results. Some of the occupations we identified may be linked with the use of biocides and pesticides as well as other organohalogen exposures, such as Photographic and film processors (biocidal preservatives, organohalogens) dry cleaning and laundry occupations (cleaning solvents, fabric finishes, and contaminated clothing), painters and decorators (working with exterior paint and its removal), construction inspectors (biocidal preservatives, pesticide contaminated areas), Mechanical engineering technologists and technicians (biocidal preservatives), railway and motor transport labourers (biocidal preservatives, possible use of herbicides along tracks) and nursery and greenhouse workers (pesticides and herbicides) (see Table 4). Occupations linked to pesticides have been implicated in several studies of pancreatic cancer but with mixed results [40]. A Spanish case control study observed an elevated risk for male employed skilled workers in gardens, nurseries and vegetable or market gardens (OR = 5.62, 95% CI: 0.48–66.10) and in agricultural workers both self-employed skilled workers in agricultural activities (OR = 1.04, 95% CI: 0.58–188) and employed skilled workers in agricultural activities (OR = 2.35, 95% CI: 0.51–10.92) [53]. More recently, in a Canadian study an increased risk of pancreatic cancer in female agricultural workers (HR = 1.36, 95% CI: 1.07–1.72) was reported [46]. Laundry and dry cleaning has also appeared in several studies with a meta-risk ratio of 1.4 reported by one meta-analysis [36, 40]. Taken together these results suggest that there may be an opportunity for pancreatic cancer prevention in occupations that involve organohalogens, pesticides and perhaps biocides exposure.

## Conclusions

We know, reasonably well, from different studies done in other countries, as well as smaller studies in Canada, that between two and 10 per cent of cancers are caused by occupational exposure [28,29,39,40,53]. The current study evaluated occupational associations with history of prostate and pancreatic cancer and found significant relationships for both type of cancers. The occupations revealed here suggest possible occupational exposures or other factors which may predispose individuals to an increased risk of disease. This information is important for if we know more about the types of exposures that cause prostate and pancreatic cancer, we should be able to act on it, as occupational exposure is preventable. The challenge moving forward will be confirming these findings and making sure we have policies and legislation appropriate to the carcinogenic hazard (e.g., possible organohalogen, pesticides, or biocides exposure).

This study aimed to highlight which occupations have an increased risk of PCa or pancreatic cancer so that further investigations may be completed to more accurately identify the causative agents. Preventive and awareness educational occupation programs and strategies for risk reduction can also be applied using the hierarchy of controls for at risk occupations. Additional research on the exposures associated with each occupation, but also evaluating the association with the province of residence, would be an asset for future research.

### Study limitations

This study has some limitations. Employment history was obtained only for one point in time (June 4, 1991) and does not contain data on duration; however; occupation at the time of survey is still representative of the exposure and activities which the individual may have participated in during this time period and serves well as a proxy for exposure. Furthermore, higher age categories such as 44–64 would most likely represent a stable and long-standing job.

The data set lacks information on family history, physical activity, smoking, diet and other lifestyle factors and comorbidities which may have affected these associations. Adjusting for income and education level does account for some smoking and lifestyle factors which have their own unique cancer risk. This study did not use detailed information on cancer histology which means that aggressive and indolent forms of cancer could not be discerned. Future studies should attempt to extend the investigation to other forms of cancer. Another limitation of this study is the lack of control for ethnicity in our statistical analyses which could have affected our estimates if there a disproportionate distribution of ethnicity by occupation is present [54].

Another limitation of this study is that it did not include measures of social inequalities in cancer which are crucial public health issues affecting particularly disadvantaged individuals. For example, in Canada and USA, the incidence rates of several preventable cancers including pancreatic cancer are generally higher in the Indigenous populations than in the non-Indigenous populations [55]. For prostate cancer, however, the incidence is increased among groups with higher SES, while late-stage prostate cancer is diagnosed more frequently among groups low on SES [56]. Incidence of prostate cancer is higher, compared to other forms of cancer also, at least partially, due to detection of many, otherwise non-detectable (symptom wise), early-stage cancers which result from differential participation in cancer screening, either organized (because an individual has reached the age of screening, e.g., 50 years in most Canadian provinces) or because the opportunity is there for screening.

Socio-economic, political, legislative and technology access and development forces impact the distribution of cancer risk factors in a population, and affect access to health services, which subsequently translate in specific observed inequalities in cancer risk and outcomes. Not included in this study are also psychosocial factors, such as mental health, known to lead in the adoption of unhealthy behaviours such as substance use, poor diet and lifestyle factors (e.g., poor sleeping and eating habits) and the exposure to a greater variety and intensity of cancer risk factors than their counterparts.

Lastly, while statistically significant, a number of occupations we examined have a CI that approach 1.0 (Tables 1 & 2). Since there is a tenuous link discussed in the introduction to chemical exposures that is not analyzed in this study, it might be reasonable to infer that these exposures are at the route of the cancer diagnosis. However, it is likely that the link is much more complex. To further understand these associations, high quality data on populations within populations are needed. Future studies should attempt to link Human Development Index, a proxy for the socioeconomic development of a country, with the magnitude and profile of pancreatic and prostate cancer to explain the distribution of cancer risk at the national level. Future studies should examine population data within populations, and stratify their analyses based on this information. Multisectoral action (socioeconomic, political, legislative) may be required to find effective solutions to the prevailing inequalities in cancer risk [57].

There are several strengths to this study. This study utilizes the largest dataset available for the Canadian population. This allowed for large sample sizes for history of prostate cancer (n = 28,610) and pancreatic cancer (n = 3,220) to be analyzed. The study also adjusted for several variables including age, sex, province of residence, income, education level, immigration status and occupation. This level of analysis also helps to account for differences in socioeconomic status and various lifestyle factors.

Furthermore, in Canada, labour laws and healthcare provisions may differ from other regions, making existing literature unreliable for our unique population. Provinces vary in age distribution, geographical factors and population size, which effects healthcare resource allotment including prevention, detection and treatment [8].

Other parameters such as marital status and ethnicity were omitted from this study but included in Sritharan et al [29]. Our study also includes occupational associations of pancreatic cancer which have not been evaluated at this scale in Canada. Furthermore, the recent publication on prostate cancer can be used as a benchmark to confirm that the models generated in this study are correct, as similar results were obtained and therefore have been included for completeness.

## Supporting information

S1 Table List of the NOC coding structure included in the study. (PDF)
