# Supplementary material for: An examination of the association between lifetime history of prostate and pancreatic cancer diagnosis and occupation in a population sample of Canadians
Source: PLoS One. 2020 Feb 5;15(2):e0227622. doi: 10.1371/journal.pone.0227622 (PMC7001921; doi:10.1371/journal.pone.0227622)
Supplement: S1 Table — (PDF) [file pone.0227622.s001.pdf]

NOC major categories

0	Management occupations
1	Business, finance and administration occupations
2	Natural and applied sciences and related occupations
3	Health occupations
4	Occupations in education, law and social, community and government services
5	Occupations in art, culture, recreation and sport
6	Sales and service occupations
7	Trades, transport and equipment operators and related occupations
8	Natural resources, agriculture and related production occupations
9	Occupations in manufacturing and utilities

NOC 2-digit coding

00	Senior management occupations
01-05	Specialized middle management occupations
06	Middle management occupations in retail and wholesale trade and customer services
07-09	Middle management occupations in trades, transportation, production and utilities
11	Professional occupations in business and finance
12	Administrative and financial supervisors and administrative occupations
13	Finance, insurance and related business administrative occupations
14	Office support workers
15	Distribution, tracking and scheduling co-ordination occupations
21	Professional occupations in natural and applied sciences
22	Technical occupations related to natural and applied sciences
30	Professional occupations in nursing
31	Professional occupations in health (except nursing)
32	Technical occupations in health
34	Assisting occupations in support of health services
40	Professional occupations in education services
41	Professional occupations in law and social, community and government services
42	Paraprofessional occupations in legal, social, community and education services
43	Occupations in front-line public protection services
44	Care providers and educational, legal and public protection support occupations
51	Professional occupations in art and culture
52	Technical occupations in art, culture, recreation and sport
62	Retail sales supervisors and specialized sales occupations
63	Service supervisors and specialized service occupations
64	Sales representatives and salespersons - wholesale retail trade
65	Service representatives and other customer and personal services occupations
66	Sales support occupations
67	Service support and other service occupations, n.e.c.

72	Industrial, electrical and construction trades
73	Maintenance and equipment operation trades
74	Other installers, repairers and servicers and material handlers
75	Transport and heavy equipment operation and related maintenance occupations
76	Trades helpers, construction labourers and related occupations
82	Supervisors and technical occupations in natural resources, agriculture and related production
84	Workers in natural resources, agriculture and related production
86	Harvesting, landscaping and natural resources labourers
92	Processing, manufacturing and utilities supervisors
94	Processing and manufacturing machine operators and related production workers
95	Assemblers in manufacturing
96	Labourers in processing, manufacturing and utilities

NOC 4-digit coding

1	0011	Legislators
2	0012	Senior government managers and officials
3	0013	Senior managers - financial, communications and other business services
4	0014	Senior managers - health, education, social and community services and membership organizations
5	0015	Senior managers - trade, broadcasting and other services, n.e.c.
6	0016	Senior managers - construction, transportation, production and utilities
7	0111	Financial managers
8	0112	Human resources managers
9	0113	Purchasing managers
10	0114	Other administrative services managers
11	0121	Insurance, real estate and financial brokerage managers
12	0122	Banking, credit and other investment managers
13	0124	Advertising, marketing and public relations managers
14	0125	Other business services managers
15	0131	Telecommunication carriers managers
16	0132	Postal and courier services managers
17	0211	Engineering managers
18	0212	Architecture and science managers
19	0213	Computer and information systems managers
20	0311	Managers in health care
21	0411	Government managers - health and social policy development and program administration
22	0412	Government managers - economic analysis, policy development and program administration
23	0413	Government managers - education policy development and program administration
24	0414	Other managers in public administration
25	0421	Administrators - post-secondary education and vocational training

26	0422	School principals and administrators of elementary and secondary education
27	0423	Managers in social, community and correctional services
28	0431	Commissioned police officers
29	0432	Fire chiefs and senior firefighting officers
30	0433	Commissioned officers of the Canadian Armed Forces
31	0511	Library, archive, museum and art gallery managers
32	0512	Managers - publishing, motion pictures, broadcasting and performing arts
33	0513	Recreation, sports and fitness program and service directors
34	0601	Corporate sales managers
35	0621	Retail and wholesale trade managers
36	0631	Restaurant and food service managers
37	0632	Accommodation service managers
38	0651	Managers in customer and personal services, n.e.c.
39	0711	Construction managers
40	0712	Home building and renovation managers
41	0714	Facility operation and maintenance managers
42	0731	Managers in transportation
43	0811	Managers in natural resources production and fishing
44	0821	Managers in agriculture
45	0822	Managers in horticulture
46	0823	Managers in aquaculture
47	0911	Manufacturing managers
48	0912	Utilities managers
49	1111	Financial auditors and accountants
50	1112	Financial and investment analysts
51	1113	Securities agents, investment dealers and brokers
52	1114	Other financial officers
53	1121	Human resources professionals
54	1122	Professional occupations in business management consulting
55	1123	Professional occupations in advertising, marketing and public relations
56	1211	Supervisors, general office and administrative support workers
57	1212	Supervisors, finance and insurance office workers
58	1213	Supervisors, library, correspondence and related information workers
59	1214	Supervisors, mail and message distribution occupations
60	1215	Supervisors, supply chain, tracking and scheduling co-ordination occupations
61	1221	Administrative officers
62	1222	Executive assistants
63	1223	Human resources and recruitment officers
64	1224	Property administrators
65	1225	Purchasing agents and officers
66	1226	Conference and event planners
67	1227	Court officers and justices of the peace
68	1228	Employment insurance, immigration, border services and revenue officers

69	1241	Administrative assistants
70	1242	Legal administrative assistants
71	1243	Medical administrative assistants
72	1251	Court reporters, medical transcriptionists and related occupations
73	1252	Health information management occupations
74	1253	Records management technicians
75	1254	Statistical officers and related research support occupations
76	1311	Accounting technicians and bookkeepers
77	1312	Insurance adjusters and claims examiners
78	1313	Insurance underwriters
79	1314	Assessors, valuators and appraisers
80	1315	Customs, ship and other brokers
81	1411	General office support workers
82	1414	Receptionists
83	1415	Personnel clerks
84	1416	Court clerks
85	1422	Data entry clerks
86	1423	Desktop publishing operators and related occupations
87	1431	Accounting and related clerks
88	1432	Payroll administrators
89	1434	Banking, insurance and other financial clerks
90	1435	Collectors
91	1451	Library assistants and clerks
92	1452	Correspondence, publication and regulatory clerks
93	1454	Survey interviewers and statistical clerks
94	1511	Mail, postal and related workers
95	1512	Letter carriers
96	1513	Couriers, messengers and door-to-door distributors
97	1521	Shippers and receivers
98	1522	Storekeepers and partspersons
99	1523	Production logistics co-ordinators
100	1524	Purchasing and inventory control workers
101	1525	Dispatchers
102	1526	Transportation route and crew schedulers
103	2111	Physicists and astronomers
104	2112	Chemists
105	2113	Geoscientists and oceanographers
106	2114	Meteorologists and climatologists
107	2115	Other professional occupations in physical sciences
108	2121	Biologists and related scientists
109	2122	Forestry professionals
110	2123	Agricultural representatives, consultants and specialists
111	2131	Civil engineers

112	2132	Mechanical engineers
113	2133	Electrical and electronics engineers
114	2134	Chemical engineers
115	2141	Industrial and manufacturing engineers
116	2142	Metallurgical and materials engineers
117	2143	Mining engineers
118	2144	Geological engineers
119	2145	Petroleum engineers
120	2146	Aerospace engineers
121	2147	Computer engineers (except software engineers and designers)
122	2148	Other professional engineers, n.e.c.
123	2151	Architects
124	2152	Landscape architects
125	2153	Urban and land use planners
126	2154	Land surveyors
127	2161	Mathematicians, statisticians and actuaries
128	2171	Information systems analysts and consultants
129	2172	Database analysts and data administrators
130	2173	Software engineers and designers
131	2174	Computer programmers and interactive media developers
132	2175	Web designers and developers
133	2211	Chemical technologists and technicians
134	2212	Geological and mineral technologists and technicians
135	2221	Biological technologists and technicians
136	2222	Agricultural and fish products inspectors
137	2223	Forestry technologists and technicians
138	2224	Conservation and fishery officers
139	2225	Landscape and horticulture technicians and specialists
140	2231	Civil engineering technologists and technicians
141	2232	Mechanical engineering technologists and technicians
142	2233	Industrial engineering and manufacturing technologists and technicians
143	2234	Construction estimators
144	2241	Electrical and electronics engineering technologists and technicians
145	2242	Electronic service technicians (household and business equipment)
146	2243	Industrial instrument technicians and mechanics
147	2244	Aircraft instrument, electrical and avionics mechanics, technicians and inspectors
148	2251	Architectural technologists and technicians
149	2252	Industrial designers
150	2253	Drafting technologists and technicians
151	2254	Land survey technologists and technicians
152	2255	Technical occupations in geomatics and meteorology
153	2261	Non-destructive testers and inspection technicians
154	2262	Engineering inspectors and regulatory officers

155	2263	Inspectors in public and environmental health and occupational health and safety
156	2264	Construction inspectors
157	2271	Air pilots, flight engineers and flying instructors
158	2272	Air traffic controllers and related occupations
159	2273	Deck officers, water transport
160	2274	Engineer officers, water transport
161	2275	Railway traffic controllers and marine traffic regulators
162	2281	Computer network technicians
163	2282	User support technicians
164	2283	Information systems testing technicians
165	3011	Nursing co-ordinators and supervisors
166	3012	Registered nurses and registered psychiatric nurses
167	3111	Specialist physicians
168	3112	General practitioners and family physicians
169	3113	Dentists
170	3114	Veterinarians
171	3121	Optometrists
172	3122	Chiropractors
173	3124	Allied primary health practitioners
174	3125	Other professional occupations in health diagnosing and treating
175	3131	Pharmacists
176	3132	Dietitians and nutritionists
177	3141	Audiologists and speech-language pathologists
178	3142	Physiotherapists
179	3143	Occupational therapists
180	3144	Other professional occupations in therapy and assessment
181	3211	Medical laboratory technologists
182	3212	Medical laboratory technicians and pathologists' assistants
183	3213	Animal health technologists and veterinary technicians
184	3214	Respiratory therapists, clinical perfusionists and cardiopulmonary technologists
185	3215	Medical radiation technologists
186	3216	Medical sonographers
187	3217	Cardiology technologists and electrophysiological diagnostic technologists, n.e.c.
188	3219	Other medical technologists and technicians (except dental health)
189	3221	Denturists
190	3222	Dental hygienists and dental therapists
191	3223	Dental technologists, technicians and laboratory assistants
192	3231	Opticians
193	3232	Practitioners of natural healing
194	3233	Licensed practical nurses
195	3234	Paramedical occupations
196	3236	Massage therapists
197	3237	Other technical occupations in therapy and assessment

198	3411	Dental assistants
199	3413	Nurse aides, orderlies and patient service associates
200	3414	Other assisting occupations in support of health services
201	4011	University professors and lecturers
202	4012	Post-secondary teaching and research assistants
203	4021	College and other vocational instructors
204	4031	Secondary school teachers
205	4032	Elementary school and kindergarten teachers
206	4033	Educational counsellors
207	4111	Judges
208	4112	Lawyers and Quebec notaries
209	4151	Psychologists
210	4152	Social workers
211	4153	Family, marriage and other related counsellors
212	4154	Professional occupations in religion
213	4155	Probation and parole officers and related occupations
214	4156	Employment counsellors
215	4161	Natural and applied science policy researchers, consultants and program officers
216	4162	Economists and economic policy researchers and analysts
217	4163	Business development officers and marketing researchers and consultants
218	4164	Social policy researchers, consultants and program officers
219	4165	Health policy researchers, consultants and program officers
220	4166	Education policy researchers, consultants and program officers
221	4167	Recreation, sports and fitness policy researchers, consultants and program officers
222	4168	Program officers unique to government
223	4169	Other professional occupations in social science, n.e.c.
224	4211	Paralegal and related occupations
225	4212	Social and community service workers
226	4214	Early childhood educators and assistants
227	4215	Instructors of persons with disabilities
228	4216	Other instructors
229	4217	Other religious occupations
230	4311	Police officers (except commissioned)
231	4312	Firefighters
232	4313	Non-commissioned ranks of the Canadian Armed Forces
233	4411	Home child care providers
234	4412	Home support workers, housekeepers and related occupations
235	4413	Elementary and secondary school teacher assistants
236	4421	Sheriffs and bailiffs
237	4422	Correctional service officers
238	4423	By-law enforcement and other regulatory officers, n.e.c.
239	5111	Librarians
240	5112	Conservators and curators

241	5113	Archivists
242	5121	Authors and writers
243	5122	Editors
244	5123	Journalists
245	5125	Translators, terminologists and interpreters
246	5131	Producers, directors, choreographers and related occupations
247	5132	Conductors, composers and arrangers
248	5133	Musicians and singers
249	5134	Dancers
250	5135	Actors and comedians
251	5136	Painters, sculptors and other visual artists
252	5211	Library and public archive technicians
253	5212	Technical occupations related to museums and art galleries
254	5221	Photographers
255	5222	Film and video camera operators
256	5223	Graphic arts technicians
257	5224	Broadcast technicians
258	5225	Audio and video recording technicians
259	5226	Other technical and co-ordinating occupations in motion pictures, broadcasting and the performing arts
260	5227	Support occupations in motion pictures, broadcasting, photography and the performing arts
261	5231	Announcers and other broadcasters
262	5232	Other performers, n.e.c.
263	5241	Graphic designers and illustrators
264	5242	Interior designers and interior decorators
265	5243	Theatre, fashion, exhibit and other creative designers
266	5244	Artisans and craftspersons
267	5245	Patternmakers - textile, leather and fur products
268	5251	Athletes
269	5252	Coaches
270	5253	Sports officials and referees
271	5254	Program leaders and instructors in recreation, sport and fitness
272	6211	Retail sales supervisors
273	6221	Technical sales specialists - wholesale trade
274	6222	Retail and wholesale buyers
275	6231	Insurance agents and brokers
276	6232	Real estate agents and salespersons
277	6235	Financial sales representatives
278	6311	Food service supervisors
279	6312	Executive housekeepers
280	6313	Accommodation, travel, tourism and related services supervisors
281	6314	Customer and information services supervisors

282	6315	Cleaning supervisors
283	6316	Other services supervisors
284	6321	Chefs
285	6322	Cooks
286	6331	Butchers, meat cutters and fishmongers - retail and wholesale
287	6332	Bakers
288	6341	Hairstylists and barbers
289	6342	Tailors, dressmakers, furriers and milliners
290	6343	Shoe repairers and shoemakers
291	6344	Jewellers, jewellery and watch repairers and related occupations
292	6345	Upholsterers
293	6346	Funeral directors and embalmers
294	6411	Sales and account representatives - wholesale trade (non-technical)
295	6421	Retail salespersons
296	6511	Maîtres d'hôtel and hosts/hostesses
297	6512	Bartenders
298	6513	Food and beverage servers
299	6521	Travel counsellors
300	6522	Pursers and flight attendants
301	6523	Airline ticket and service agents
302	6524	Ground and water transport ticket agents, cargo service representatives and related clerks
303	6525	Hotel front desk clerks
304	6531	Tour and travel guides
305	6532	Outdoor sport and recreational guides
306	6533	Casino occupations
307	6541	Security guards and related security service occupations
308	6551	Customer services representatives - financial institutions
309	6552	Other customer and information services representatives
310	6561	Image, social and other personal consultants
311	6562	Estheticians, electrologists and related occupations
312	6563	Pet groomers and animal care workers
313	6564	Other personal service occupations
314	6611	Cashiers
315	6621	Service station attendants
316	6622	Store shelf stockers, clerks and order fillers
317	6623	Other sales related occupations
318	6711	Food counter attendants, kitchen helpers and related support occupations
319	6721	Support occupations in accommodation, travel and facilities set-up services
320	6722	Operators and attendants in amusement, recreation and sport
321	6731	Light duty cleaners
322	6732	Specialized cleaners
323	6733	Janitors, caretakers and building superintendents

324	6741	Dry cleaning, laundry and related occupations
325	6742	Other service support occupations, n.e.c.
326	7201	Contractors and supervisors, machining, metal forming, shaping and erecting trades and related occupations
327	7202	Contractors and supervisors, electrical trades and telecommunications occupations
328	7203	Contractors and supervisors, pipefitting trades
329	7204	Contractors and supervisors, carpentry trades
330	7205	Contractors and supervisors, other construction trades, installers, repairers and servicers
331	7231	Machinists and machining and tooling inspectors
332	7232	Tool and die makers
333	7233	Sheet metal workers
334	7234	Boilermakers
335	7235	Structural metal and platework fabricators and fitters
336	7236	Ironworkers
337	7237	Welders and related machine operators
338	7241	Electricians (except industrial and power system)
339	7242	Industrial electricians
340	7243	Power system electricians
341	7244	Electrical power line and cable workers
342	7245	Telecommunications line and cable workers
343	7246	Telecommunications installation and repair workers
344	7247	Cable television service and maintenance technicians
345	7251	Plumbers
346	7252	Steamfitters, pipefitters and sprinkler system installers
347	7253	Gas fitters
348	7271	Carpenters
349	7272	Cabinetmakers
350	7281	Bricklayers
351	7282	Concrete finishers
352	7283	Tilesetters
353	7284	Plasterers, drywall installers and finishers and lathers
354	7291	Roofers and shinglers
355	7292	Glaziers
356	7293	Insulators
357	7294	Painters and decorators (except interior decorators)
358	7295	Floor covering installers
359	7301	Contractors and supervisors, mechanic trades
360	7302	Contractors and supervisors, heavy equipment operator crews
361	7303	Supervisors, printing and related occupations
362	7304	Supervisors, railway transport operations
363	7305	Supervisors, motor transport and other ground transit operators
364	7311	Construction millwrights and industrial mechanics

365	7312	Heavy-duty equipment mechanics
366	7313	Heating, refrigeration and air conditioning mechanics
367	7314	Railway carmen/women
368	7315	Aircraft mechanics and aircraft inspectors
369	7316	Machine fitters
370	7318	Elevator constructors and mechanics
371	7321	Automotive service technicians, truck and bus mechanics and mechanical repairers
372	7322	Motor vehicle body repairers
373	7331	Oil and solid fuel heating mechanics
374	7332	Appliance servicers and repairers
375	7333	Electrical mechanics
376	7334	Motorcycle, all-terrain vehicle and other related mechanics
377	7335	Other small engine and small equipment repairers
378	7361	Railway and yard locomotive engineers
379	7362	Railway conductors and brakemen/women
380	7371	Crane operators
381	7372	Drillers and blasters - surface mining, quarrying and construction
382	7373	Water well drillers
383	7381	Printing press operators
384	7384	Other trades and related occupations, n.e.c.
385	7441	Residential and commercial installers and servicers
386	7442	Waterworks and gas maintenance workers
387	7444	Pest controllers and fumigators
388	7445	Other repairers and servicers
389	7451	Longshore workers
390	7452	Material handlers
391	7511	Transport truck drivers
392	7512	Bus drivers, subway operators and other transit operators
393	7513	Taxi and limousine drivers and chauffeurs
394	7514	Delivery and courier service drivers
395	7521	Heavy equipment operators (except crane)
396	7522	Public works maintenance equipment operators and related workers
397	7531	Railway yard and track maintenance workers
398	7532	Water transport deck and engine room crew
399	7533	Boat and cable ferry operators and related occupations
400	7534	Air transport ramp attendants
401	7535	Other automotive mechanical installers and servicers
402	7611	Construction trades helpers and labourers
403	7612	Other trades helpers and labourers
404	7621	Public works and maintenance labourers
405	7622	Railway and motor transport labourers
406	8211	Supervisors, logging and forestry
407	8221	Supervisors, mining and quarrying

408	8222	Contractors and supervisors, oil and gas drilling and services
409	8231	Underground production and development miners
410	8232	Oil and gas well drillers, servicers, testers and related workers
411	8241	Logging machinery operators
412	8252	Agricultural service contractors, farm supervisors and specialized livestock workers
413	8255	Contractors and supervisors, landscaping, grounds maintenance and horticulture services
414	8261	Fishing masters and officers
415	8262	Fishermen/women
416	8411	Underground mine service and support workers
417	8412	Oil and gas well drilling and related workers and services operators
418	8421	Chain saw and skidder operators
419	8422	Silviculture and forestry workers
420	8431	General farm workers
421	8432	Nursery and greenhouse workers
422	8441	Fishing vessel deckhands
423	8442	Trappers and hunters
424	8611	Harvesting labourers
425	8612	Landscaping and grounds maintenance labourers
426	8613	Aquaculture and marine harvest labourers
427	8614	Mine labourers
428	8615	Oil and gas drilling, servicing and related labourers
429	8616	Logging and forestry labourers
430	9211	Supervisors, mineral and metal processing
431	9212	Supervisors, petroleum, gas and chemical processing and utilities
432	9213	Supervisors, food and beverage processing
433	9214	Supervisors, plastic and rubber products manufacturing
434	9215	Supervisors, forest products processing
435	9217	Supervisors, textile, fabric, fur and leather products processing and manufacturing
436	9221	Supervisors, motor vehicle assembling
437	9222	Supervisors, electronics manufacturing
438	9223	Supervisors, electrical products manufacturing
439	9224	Supervisors, furniture and fixtures manufacturing
440	9226	Supervisors, other mechanical and metal products manufacturing
441	9227	Supervisors, other products manufacturing and assembly
442	9231	Central control and process operators, mineral and metal processing
443	9232	Central control and process operators, petroleum, gas and chemical processing
444	9235	Pulping, papermaking and coating control operators
445	9241	Power engineers and power systems operators
446	9243	Water and waste treatment plant operators
447	9411	Machine operators, mineral and metal processing
448	9412	Foundry workers
449	9413	Glass forming and finishing machine operators and glass cutters

450	9414	Concrete, clay and stone forming operators
451	9415	Inspectors and testers, mineral and metal processing
452	9416	Metalworking and forging machine operators
453	9417	Machining tool operators
454	9418	Other metal products machine operators
455	9421	Chemical plant machine operators
456	9422	Plastics processing machine operators
457	9423	Rubber processing machine operators and related workers
458	9431	Sawmill machine operators
459	9432	Pulp mill machine operators
460	9433	Papermaking and finishing machine operators
461	9434	Other wood processing machine operators
462	9435	Paper converting machine operators
463	9436	Lumber graders and other wood processing inspectors and graders
464	9437	Woodworking machine operators
465	9441	Textile fibre and yarn, hide and pelt processing machine operators and workers
466	9442	Weavers, knitters and other fabric making occupations
467	9445	Fabric, fur and leather cutters
468	9446	Industrial sewing machine operators
469	9447	Inspectors and graders, textile, fabric, fur and leather products manufacturing
470	9461	Process control and machine operators, food and beverage processing
471	9462	Industrial butchers and meat cutters, poultry preparers and related workers
472	9463	Fish and seafood plant workers
473	9465	Testers and graders, food and beverage processing
474	9471	Plateless printing equipment operators
475	9472	Camera, platemaking and other prepress occupations
476	9473	Binding and finishing machine operators
477	9474	Photographic and film processors
478	9521	Aircraft assemblers and aircraft assembly inspectors
479	9522	Motor vehicle assemblers, inspectors and testers
480	9523	Electronics assemblers, fabricators, inspectors and testers
481	9524	Assemblers and inspectors, electrical appliance, apparatus and equipment manufacturing
482	9525	Assemblers, fabricators and inspectors, industrial electrical motors and transformers
483	9526	Mechanical assemblers and inspectors
484	9527	Machine operators and inspectors, electrical apparatus manufacturing
485	9531	Boat assemblers and inspectors
486	9532	Furniture and fixture assemblers and inspectors
487	9533	Other wood products assemblers and inspectors
488	9534	Furniture finishers and refinishers
489	9535	Plastic products assemblers, finishers and inspectors
490	9536	Industrial painters, coaters and metal finishing process operators

491	9537	Other products assemblers, finishers and inspectors
492	9611	Labourers in mineral and metal processing
493	9612	Labourers in metal fabrication
494	9613	Labourers in chemical products processing and utilities
495	9614	Labourers in wood, pulp and paper processing
496	9615	Labourers in rubber and plastic products manufacturing
497	9616	Labourers in textile processing
498	9617	Labourers in food and beverage processing
499	9618	Labourers in fish and seafood processing
500	9619	Other labourers in processing, manufacturing and utilities
